# 181. Potential Benefit of Masking and other COVID-19 Infection Prevention Measures on Late-Onset Infections in the NICU

**DOI:** 10.1093/ofid/ofab466.181

**Published:** 2021-12-04

**Authors:** Charlene Bultmann, Jaclyn Wiggins, Sagori Mukhopadhyay, Dustin Flannery, Mark Conaway, Miren Dhudasia, Sam Garber, Joseph B Cantey, Robert Schelonka, H Weitkamp, Kristin Weimer, Dipen Vyas, Margaret Gilfillan, Alison Carey, Julie Wohrley, Andrew Berenz, Sarah Khan, M Favara, D Tuttle, K Ziegler, E Chang, J Gaulton, Pablo J Sanchez, David Kaufman

**Affiliations:** 1 University of Virginia, Charlottesville, Virginia; 2 UVA, Charlottesville, Virginia; 3 U PENN, Philadelphia, Pennsylvania; 4 UPENN, Phila, Pennsylvania; 5 DUKE, durham, North Carolina; 6 University of Texas Health San Antonio, San Antonio, TX; 7 Oregon, Oregan, Oregon; 8 Vanderbilt, Nashville, Tennessee; 9 St Chris, PHila, Pennsylvania; 10 St Christopher’s, Phila, Pennsylvania; 11 Rush, Chicago, Illinois; 12 McMaster University, Hamilton, Ontario, Canada, Hamilton, Ontario, Canada; 13 Christiana, Wilmington, Delaware; 14 Abington, Abington, Pennsylvania; 15 Nationwide Children’s Hospital - The Ohio State University, Columbus, Ohio

## Abstract

**Background:**

Incidence of blood stream infections (BSI) among NICU admissions remains high, with associated mortality and morbidity. Due to COVID-19, there are increased infection prevention (IP) measures in NICUs including universal masking for all healthcare workers and families, social distancing, visitation restrictions, and increased attention to hand hygiene. These measures may also affect late-onset infection rates and offer understanding of novel interventions for prevention.

**Methods:**

We examined infection rates during the 24 months prior to implementation of COVID-19 IP measures (PRE-period) compared to the months after implementation from April 2020 (POST-period). Late-onset infections were defined as culture-confirmed infection of the blood, urine, or identification of respiratory viral pathogens. An interrupted time series analysis of infection per 1000 patient days was performed based on a change-point Poisson regression with a lagged dependent variable and the number of patient days used as offsets. Each month was treated as independent with additional analysis using an observation-driven model to account for serial dependence.

**Results:**

Multicenter analysis to date included all infants cared for at three centers (Level 3 and 4) from 2018-2020. Monthly BSI rates decreased in the POST-period at the three centers (**Figure 1). ** At all centers actual BSI rate was lower than the expected rate in the POST-period (**Figure 2).** The combined BSI rate per 1000 patient days was 41% lower compared to the rate prior to implementation (95% CI, 0.42 to 0.84, P=0.004) (**Table 1**). In subgroup analysis by birthweight, infants< 1000g had a 39% reduction in BSI (P=0.023), for1000-1500g patients there was a 44% reduction (P=0.292) and in those > 1500g there was a 53% reduction (0.083).

Figure 1. PRE and POST MASKING and other COVID Infection Prevention Measures and Monthly BSI Rates.

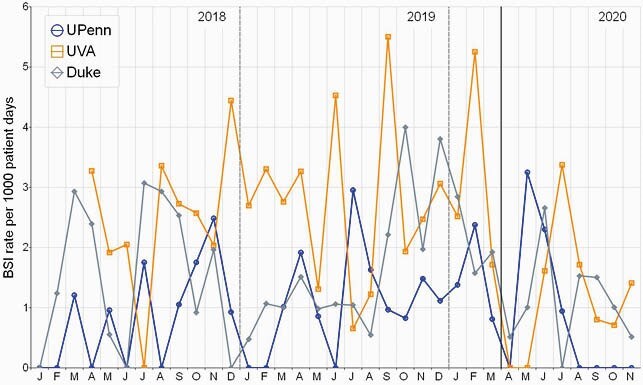

Figure 2. PRE and POST MASKING and other COVID infection prevention measures and BSI Trends.

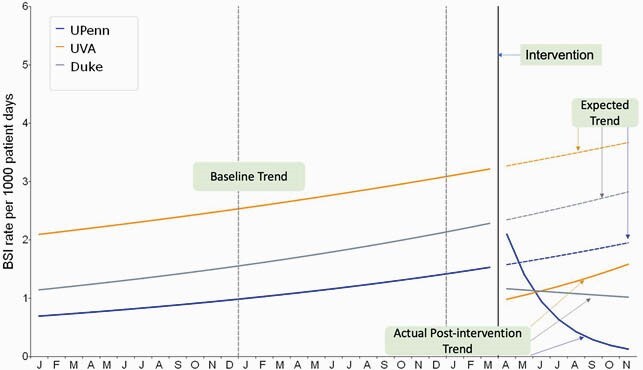

At all centers actual BSI rate was lower than the expected rate for that center in the POST period. UVA and Duke showed a baseline decrease and Pennsylvania Hospital showed a downward trend in infection rates. There was an approximate decrease in expected bloodstream infection events at Pennsylvania Hospital by 7 events, at UVA by 22 events and at Duke by 23 events. Overall, all three centers saw a decrease in their expected infections after COVID-19 infection prevention measures were implemented.

Table 1. Percent reduction in Bloodstream Infection at each center.

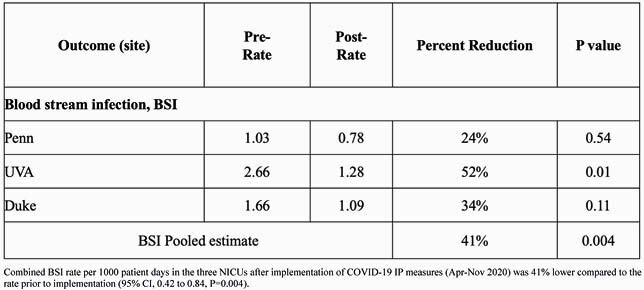

**Conclusion:**

In this preliminary analysis, we found a reduction of BSI after the implementation of COVID-19 infection prevention measures. Additionally, there were fewer viral infections, though there were a limited number of episodes. Further analyses of multicenter data and a larger number of patients will elucidate the significance of these findings and the role some of these IP measures such as universal masking may have in infection prevention in the NICU.

**Disclosures:**

**All Authors**: No reported disclosures

